# Intraoral Condylectomy with 3D-Printed Cutting Guide versus with Surgical Navigation: An Accuracy and Effectiveness Comparison

**DOI:** 10.3390/jcm12113816

**Published:** 2023-06-02

**Authors:** Jiawen Si, Chenglong Zhang, Ming Tian, Tengfei Jiang, Lei Zhang, Hongbo Yu, Jun Shi, Xudong Wang

**Affiliations:** Department of Oral and Craniomaxillofacial Surgery, Shanghai Ninth People’s Hospital, College of Stomatology, Shanghai Jiao Tong University School of Medicine, National Clinical Research Center for Oral Diseases, Shanghai Key Laboratory of Stomatology, Shanghai Research Institute of Stomatology, No. 639, Zhizaoju Road, Shanghai 200011, China

**Keywords:** condylar osteochondroma, intraoral condylectomy, 3D printing, titanium cutting guide, surgical navigation

## Abstract

This study compares the accuracy and effectiveness of our novel 3D-printed titanium cutting guides with intraoperative surgical navigation for performing intraoral condylectomy in patients with mandibular condylar osteochondroma (OC). A total of 21 patients with mandibular condylar OC underwent intraoral condylectomy with either 3D-printed cutting guides (cutting guide group) or with surgical navigation (navigation group). The condylectomy accuracy in the cutting guide group and navigation group was determined by analyzing the three-dimensional (3D) discrepancies between the postoperative computed tomography (CT) images and the preoperative virtual surgical plan (VSP). Moreover, the improvement of the mandibular symmetry in both groups was determined by evaluating the chin deviation, chin rotation and mandibular asymmetry index (AI). The superimposition of the condylar osteotomy area showed that the postoperative results were very close to the VSP in both groups. The mean 3D deviation and maximum 3D deviation between the planned condylectomy and the actual result were 1.20 ± 0.60 mm and 2.36 ± 0.51 mm in the cutting guide group, and 1.33 ± 0.76 mm and 4.27 ± 1.99 mm in the navigation group. Moreover, the facial symmetry was greatly improved in both groups, indicated by significantly decreased chin deviation, chin rotation and AI. In conclusion, our results show that both 3D-printed cutting-guide-assisted and surgical-navigation-assisted methods of intraoral condylectomy have high accuracy and efficiency, while using a cutting guide can generate a relatively higher surgical accuracy. Moreover, our cutting guides exhibit user-friendly features and simplicity, which represents a promising prospect in everyday clinical practice.

## 1. Introduction

Osteochondroma (OC) of the craniofacial region is a rare benign pathologic condition, which occurs primarily in the mandibular condyle and coronoid process [1]. As condylar OC is progressive and can cause severe facial deformity and oral functional disturbances, treatment usually involves a condylectomy [2,3,4]. With the recently fast-developed and clinically broadened application of computer-assisted surgery (CAS), the availability of various sophisticated CAS techniques has significantly grown in the maxillofacial surgery field, such as computer-assisted surgical planning, surgical navigation of jaw bone resection, augmented or mixed reality in combination with surgical navigation, and rapid prototyping of patient-specific surgical guides and fixation plates [5,6,7,8,9,10,11,12,13].

Notably, both surgical navigation and 3D-printed cutting guides have been successfully applied in condylectomy. Huo et al. [7] fabricated a patient-specific surgical cutting guide to pilot the endoscopically assisted vertical ramus osteotomy for treatment of mandibular condylar osteochondroma. Haas et al. [5] introduced an intraoral proportional condylectomy guide for treatment of mandibular condylar hyperplasia. Moreover, we have successfully implemented the computerized navigation in condylectomy and gap surgery of temporomandibular joint ankylosis (TMJA), which allowed the surgeon to apply the preoperative virtual surgical plan (VSP) to the operation precisely during the procedure [14,15]. These reports indicated that the application of both individualized cutting guides and surgical navigation for various condylectomy procedures represents a promising method for the accurate reproduction of the preoperative VSP and reduction of surgical complications.

Specifically, higher technique difficulties such as insufficient intraoral visualization and resection of the tumor, which increase the risk of intraoperative injury of vital anatomic structures and the skull base, are commonly encountered when performing an intraoral condylectomy. To increase the accuracy and safety of an intraoral condylectomy, both computerized-navigation and a 3D-printed osteotomy template were proposed as feasible solutions. However, none of the published data specifically examined the accuracy of patient-specific condylar osteotomy cutting guides to compare it with a navigation-assisted condylectomy. Based on previous reports and our own experience, we recently developed a minimally invasive 3D-printed titanium cutting guide for intraoral condylectomy. In this study, we report the successful application of these guides in patients with mandibular condylar OC and evaluate the accuracy of these guides and effectiveness of the surgery in comparison with computer navigation as a proof-of-concept demonstration.

## 2. Materials and Methods

This retrospective study was conducted at the Department of Oral and Cranio-Maxillofacial Surgery in Shanghai Ninth Peoples Hospital and approved by the constitutional ethics committee (SH9H-2019-T114-1). From 2020 to 2022, 21 patients with the diagnosis of mandibular condylar osteochondroma were included in this study and gave written consent before the procedure. Of all the included patients, 10 patients underwent condylectomy assisted by the 3D-printed titanium cutting guides (cutting guide group), while 11 patients underwent condylectomy assisted by computer navigation (navigation group).

### 2.1. Computer Assisted Surgical Planning and Titanium Cutting Guide 3D Printing

**Figure 1:**
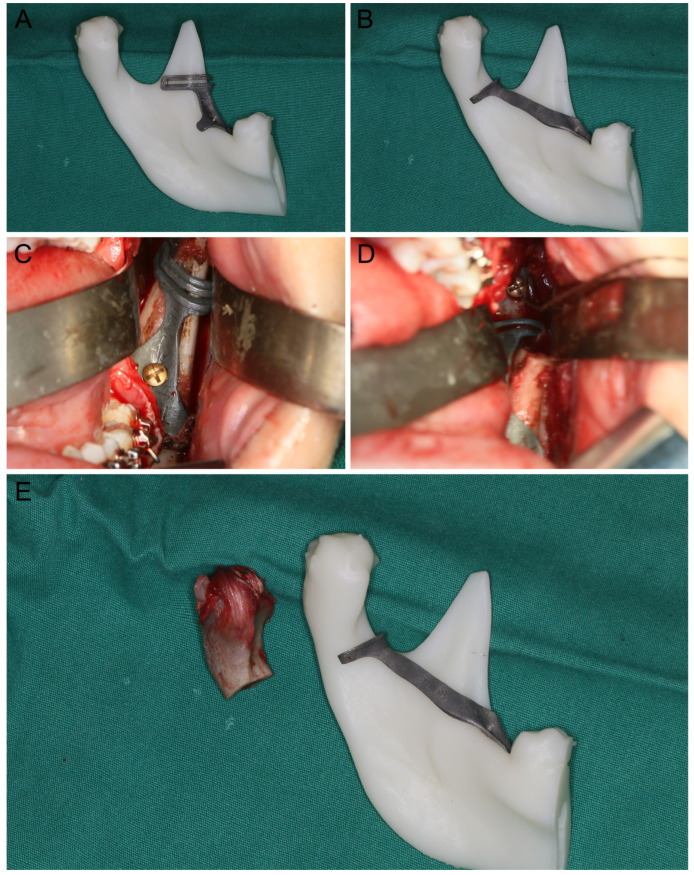
Fabrication and application of the titanium cutting guide. (**A**) 3D-printed titanium cutting guides for coronoidectomy; (**B**) 3D-printed titanium cutting guides for condylectomy; (**C**) intraoperative application of the coronoidectomy cutting guide; (**D**) intraoperative application of the condylectomy cutting guide; (**E**) removed condyle with the condylectomy cutting guide.

The virtual surgical planning and titanium cutting guide 3D printing proceeded as we previously reported [2,10]. Briefly, DICOM data files obtained from maxillofacial computed tomography (CT, LightSpeed CT scanner, GE Healthcare, Hatfield, UK) or cone-beam computed tomography (CBCT, i-CAT, Imaging Sciences International, Hatfield, PA, USA) were imported into the ProPlan CMF software (Materialise, Leuven, Belgium). The osteotomy lines of the coronoidectomy and condylectomy were delineated virtually based on multiplanar (axial, coronal, and sagittal) and three-dimensional (3D) views of the lesion. The data set of the bony segments after the osteotomy was virtually imported into Geomagic Studio 2013 Software (Geomagic, Durham, NC, USA) to design the TiAI64V coronoidectomy guide and the condylectomy guide, which were fabricated using a titanium 3D printer (M2 cusing Mutilaser; CONCEPTLASER, Schwabhausen, Germany) (Figure 1A,B).

### 2.2. Surgical Procedure

**Figure 2:**
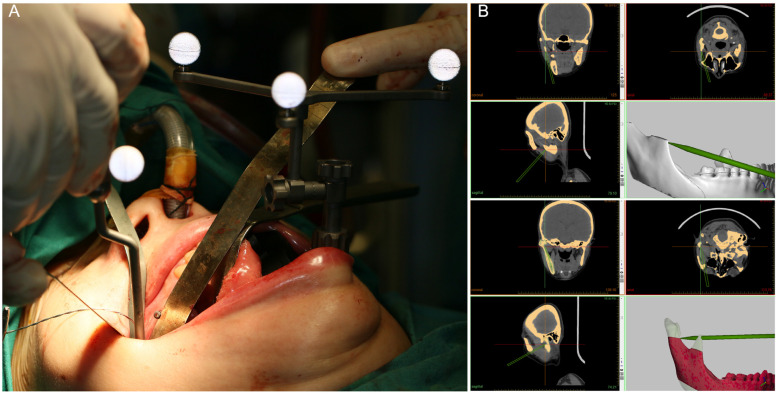
Real-time surgical visualization and intraoperative navigation guided condylectomy. (**A**) The navigation probe was used to check the accuracy of registration and locate the osteotomy plane; (**B**) the condylectomy outcomes were confirmed with real-time navigation aids by pinpointing the osteotomy plane and nearby landmarks after the surgery.

All operations were performed by the same surgical team in this study. Briefly, patients were treated with intraoral condylectomy alone or in combination with Le Fort I osteotomy and ipsilesional sagittal split ramus osteotomy. Additionally, genioplasty and contour trimming were performed to optimize the facial symmetry and achieve better esthetic outcomes. For intraoral condylectomy, a vertical incision was made in the buccal mucosa region in front of the mandibular ramus from the level of the mandibular second molar to the level of the maxillary teeth with maximum mouth opening. The buccal and lingual muco-periosteal flaps were elevated to expose the coronoid process. For the templates-guided condylectomy, the coronoidectomy guide covering the anterior border of the mandibular ramus was fixed with two temporary screws and the coronoid process was osteotomized at the level of the cutting plane using a reciprocating saw according to the presurgical plan (Figure 1C). Then, the subperiosteal dissection of the TMJ capsule and the lateral pterygoid muscle along the sigmoid notch, condylar neck and head was carried out until the condyle was fully exposed. Anatomic landmarks of the ramus–condyle unit were identified and the condylectomy guide covering the anterior border of the mandibular ramus, the sigmoid notch and the medial condylar neck region beneath the condylar osteotomy line was positioned according to its surface-best-fit and fixed with the same temporary screws (Figure 1D). Finally, the condyle was osteotomized and removed at the level of the cutting plane using a reciprocating saw according to the presurgical plan with direct vision or an endoscopic vision (Figure 1E). For navigated condylectomy, the intraoperative surgical navigation proceeded as we previously described [15,16]. Briefly, a calibrated saw with reflective balls was used to perform the condylectomy with real-time navigation aids (Figure 2A), while a navigation probe was used to ensure the favorable outcome by pinpointing the osteotomy plane and nearby landmarks during the surgery (Figure 2B).

### 2.3. Condylectomy Accuracy Validation–Evaluation of 3D Condylar Residual Deviation

**Figure 3:**
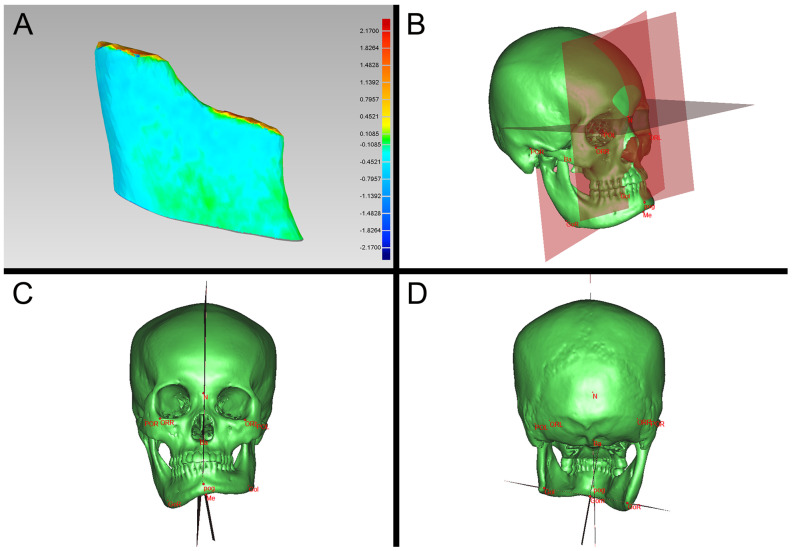
Computerized accuracy validation and effectiveness assessment. (**A**) The 3D models of virtually planned and the achieved actual residual segment of the affected condyle after condylectomy were reconstructed and imported into Geomagic Studio 2013 Software (Geomagic, Durham, NC, USA) to measure the osteotomy accuracy in terms of mean 3D deviation and maximum 3D deviation; (**B**) three reference planes and the 3D coordinate system with N points as the zero point were established on the reconstructed virtual model in the ProPlan CMF software (Materialise, Belgium) before evaluation of the mandibular symmetry; (**C**) anterior view of the mandibular plane, mandibular midsagittal plane and facial midsagittal plane; (**D**) posterior view of the mandibular plane, mandibular midsagittal plane and facial midsagittal plane.

The accuracy evaluation of the condylectomy in both the cutting guide group and the navigation group proceeded as previously described [9,10]. Briefly, a craniomaxillofacial CT scan was taken for each patient at 3 days postoperatively. The 3D models of the virtually planned and the achieved actual residual segment of the affected condyle after condylectomy were reconstructed and overlapped using a ProPlan CMF software (Materialise, Leuven, Belgium). The overlapped STL files were then imported into Geomagic Studio 2013 Software (Geomagic, Durham, NC, USA) and the deviations were measured as mean 3D deviation and maximum 3D deviation (Figure 3A).

### 2.4. Effectiveness Assessment–Evaluation of the Restoration of Mandibular Symmetry

The ProPlan CMF software (Materialise, Belgium) was used to reconstruct the 3D virtual models and evaluate the mandibular symmetry in both the cutting guide group and navigation group before and right after surgery, as we recently described [2]. Briefly, six fundamental marker points were established on the craniofacial skeleton model, namely Nasion (N), bilateral Orbitale (OrL, OrR), bilateral Porion (PoL, PoR) and Basion (Ba). Then, three reference planes and the basic coordinate system (x,y,z) with N points as the zero point were established (Figure 3B). Moreover, the Pogonion (Pog), Menton (Me), Mental foramen (MF), Sigmoid notch (Sg) and bilateral Gonions (GoL, GoR) were marked on the virtually reconstructed craniofacial skeleton model, with a landmark GoM defined at the middle of the GOL–GOR line. The mandibular plane was defined as a plane passing through Me, GoL and GoR, while the mandibular midsagittal plane was defined as a plane which was perpendicular to the mandibular plane passing through GoM and Me (Figure 3C,D). Accordingly, the chin deviation was measured as the distance from the Pog to the midsagittal mandibular plane, and the chin rotation was calculated as the angle between the facial midsagittal plane and the mandibular midsagittal plane. Subsequently, the distances from bilateral Go, MF and Sg to reference planes were defined as dx, dy, dz, with R and L representing the left and right side. Thus, the asymmetry index (AI) of the mandible indicated by Go, MF and Sg points, respectively, was calculated by a formula of √(Rdx−Ldx)^2^ + (Rdy − Ldy)^2^ + (Rdz−Ldz)^2^.

### 2.5. Statistical Analysis

SPSS version 21 (IBM, Chicago, IL, USA) and GraphPad Prism version 5 (GraphPad Software, La Jolla, CA, USA) were used for statistical analysis and graphical representation. Normalized measurement data are presented as mean ± standard deviation. Numerical data comparison was performed using dependent or independent *t* tests, where *p* < 0.05 (*) was considered significant.

## 3. Results

In total, 21 patients were included in this study. The cutting guide group comprised 10 patients, and the navigation group comprised 11 patients. No differences in patients’ backgrounds were noted. All clinical information of the patients was presented in Table 1. The mean patient age in the cutting guide group was 26.30 ± 4.24 years (range, 22–34 years), while that in the navigation group was 22.73 ± 3.66 years (range, 18–30 years) without significant difference. All 21 patients in both groups recovered well after surgery. The patients received routine postoperative follow-up to report complications, while no severe complications such as malocclusion, bad fractures, postoperative bleeding and infections, tumor recurrence, TMJ ankylosis or prolonged joint pain were noted in both groups. Notably, postoperative treatment with elastics bimaxillary traction and corrective orthodontics were inevitably needed.

**Figure 4:**
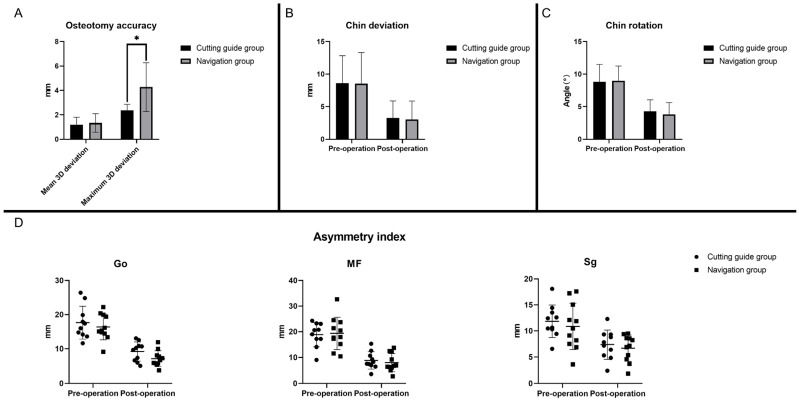
The osteotomy accuracy and mandibular symmetry assessment in both the cutting guides group and the navigation group. (**A**) The 3D deviation analysis in terms of mean 3D deviation and maximum 3D deviation in both groups; (**B**) improvement of the chin deviation in both groups after surgery; (**C**) improvement of the chin rotation in both groups after surgery; (**D**) improvement of the asymmetry index (AI) of the landmarks Go, MF and Sg in both groups after surgery. * The navigation group showed a significant higher maximum 3D deviation (*p* < 0.01).

After superimposition of the virtually planned and the achieved actual condylar residual segments, 3D osteotomy deviation analysis showed that the surgical outcome of both the cutting guide group and the navigation group exhibited a high degree of similarity to the virtual surgical planning (Figure 4A). In the cutting guide group, the mean 3D deviation of the actual residual condylar segments compared with the virtual ones was 1.20 ± 0.60 mm, while the maximum 3D deviation was 2.36 ± 0.51 mm. In the navigation group, the mean 3D deviation of the actual residual condylar segments compared with the virtual ones was 1.33 ± 0.76 mm, while the maximum 3D deviation was 4.27 ± 1.99 mm. Notably, the navigation group showed a significant higher maximum 3D deviation (*p* < 0.01).

In terms of the effectiveness of the surgery, the chin deviation, chin rotation and AI all showed significant postoperative improvements in both groups (Figure 4B–D). In more detail, the chin deviation was significantly decreased after the surgery in both the cutting guide group (from 8.61 ± 4.21 to 3.26 ± 2.62 mm, *p* = 0.0058) and the navigation group (from 8.54 ± 4.77 to 3.03 ± 2.83 mm, *p* = 0.0034), while the angle of chin rotation was significantly reduced after the surgery in both the cutting guide group (from 8.82 ± 2.67 to 4.28 ± 1.78°, *p* = 0.0004) and the navigation group (from 8.98 ± 2.28 to 3.81 ± 1.82°, *p* = 0.0001). Moreover, the asymmetry index of the landmarks Go, MF and Sg all decreased significantly (AI of Go: from 17.64 ± 4.83 to 9.21 ± 2.76 mm in the cutting guide group, from 16.37 ± 3.77 to 7.13 ± 2.28 mm in the navigation group, both *p* < 0.01; AI of MF: from 18.82 ± 4.69 to 8.83 ± 3.31 mm in the cutting guide group, from 19.31 ± 6.28 to 8.01 ± 3.48 mm in the navigation group, both *p* < 0.001; AI of Sg: from 11.83 ± 3.14 to 7.36 ± 2.79 mm in the cutting guide group, from 10.86 ± 4.43 to 6.69 ± 2.51 mm in the navigation group, both *p* < 0.05). When comparing the two groups in terms of all the measurements above, no significant difference was noted, indicating that patients in both groups exhibited great improvements in mandible symmetry (Table 2).

## 4. Discussion

For resection of the condylar OC, there are two ways of incision in the literature, namely the extraoral and intraoral approaches. The extraoral incision guarantees surgical exposure and accuracy. However, such approach might increase the risk of postoperative facial nerve injury, salivary fistula and visible facial scar formation, while the intraoral-approached condylectomy provides a more direct path to the lesion and significantly minimizes the incidence of extraoral access complications [15,17,18]. Additionally, when performed in combination with orthognathic surgery, the intraoral approach may reduce the chance of infection, because all procedures are performed through the intraoral access, avoiding intraoral and extraoral communications [5,17]. However, a procedural dilemma still existed in terms of insufficient intraoral visualization and resection of the tumor, difficulty in shaving and positioning the residual condyle, and the increasing risk of intraoperative injury of vital anatomic structures, such as the internal carotid artery, the maxillary artery, the plexus pterygoids and the skull base [5,10,18]. Thus, intraoral condylectomy needed to proceed with high accuracy and good surgical experience, which largely increased the technique difficulties and limited its popularity.

**Figure 5:**
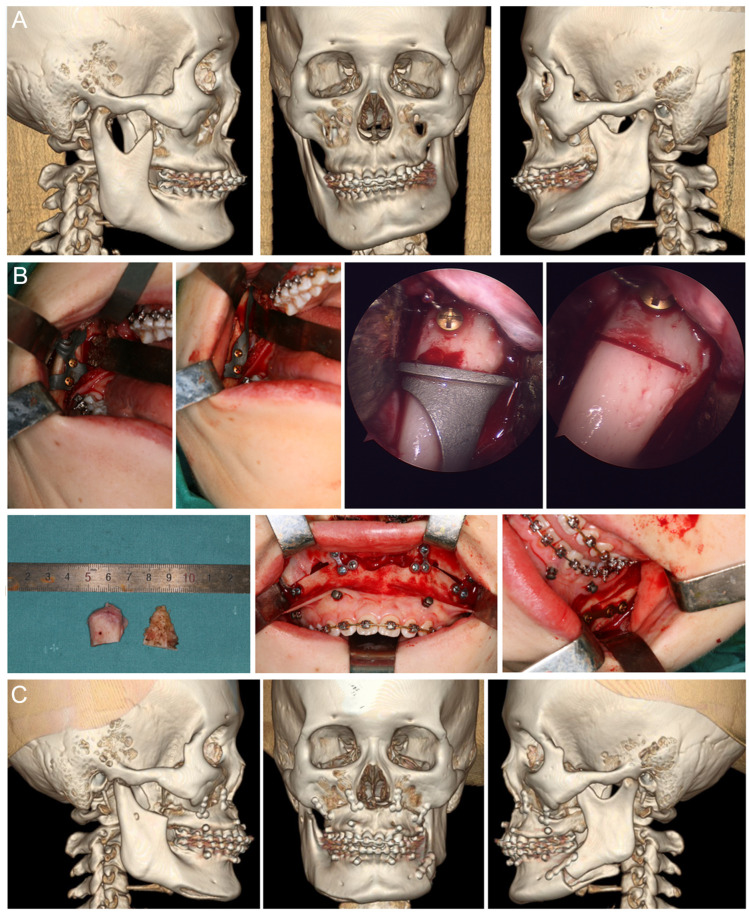
Representative mandibular OC cases treated with the 3D-printed titanium cutting guides: (**A**) preoperative CT scans showing right mandibular OC, hyperplastic mandibular body and significant chin deviation; (**B**) intraoperative application of the coronoidectomy cutting guide and condylectomy cutting guide to remove the right mandibular condyle and coronoid according to the surgical plan; moreover, Lefort I osteotomy was performed to correct the secondary maxillary deformity, sagittal split ramus osteotomy was performed on the ipsilesional side in combination with intraoral condylectomy and contour trimming to correct the mandibular asymmetry and malocclusion; (**C**) reconstruction of the postoperative CT scans showing right mandibular condyle and coronoid were accurately removed according to the surgical plan and patients asymmetry was obviously corrected.

Notably, Haas et al. [5] recently introduced an intraoral proportional condylectomy template for the treatment of mandibular condylar hyperplasia. Unlike their plastic template for proportional condylectomy, our method provides a simple way to perform intraoral total condylectomy. Since the titanium cutting template was much stronger and thinner than a plastic template, it was fixed in place with two temporary screws on the anterior mandibular ramus; our thin cutting guide can be perfectly aligned and fixed on the inferior internal side of the mandible rigidly and provide a large space, allowing osteotomy from the sigmoid notch level to the high level of condylar neck while maintaining excellent access and resection orientation during the condylectomy, having thus assured the accuracy and efficiency of the osteotomy. In more detail, the mean 3D deviation and maximum 3D deviation between the planned condylectomy and the postoperative result were 1.20 ± 0.60 mm and 2.36 ± 0.51 mm in the cutting guide group. The accuracy of both cutting guide and navigation approaches in our study was largely supported by the previous literature. In a study by Wael et al. [6], the navigated bilateral mandibulectomies and maxillectomies were performed on five cadavers and five patients with a mean cutting accuracy less than 2 mm. Moreover, mandibular osteotomies aided with 3D-printed rigid cutting guides were performed in nine patients and showed that the distance between preoperatively planned cutting plane and the performed cutting plane was 1.2 ± 1.0 mm for the anterior osteotomy and 2.2 ± 0.9 mm for the posterior osteotomy [19]. Recently, Tang et al. [11] reported the successful application of mixed reality combined with surgical navigation in mandibular tumor resection with a mean 3D-cutting deviation of 1.68 ± 0.92 mm and maximum 3D-cutting deviation of 3.46 mm. In addition, Ming Zhu et al. [13] compared the accuracy, efficacy, and safety of navigation versus individualized guides in mandibular angle osteotomy. Their results showed that the accuracies for the navigation group (1.18 ± 0.34 mm) and the guide group (0.96 ± 0.42 mm) were equivalent and both significantly higher than that of the freehand group (3.64 ± 0.77 mm). This previous literature, as well as the results in this study, indicated that our 3D-printed titanium cutting guide was highly feasible for intraoral condylectomy (Figure 5). However, for better cost savings of the surgical templates, further studies on alternative resin templates with similar or better mechanical performances are still necessary. Anna et al. [20] compared the differences between two rigid 3D printing resins (BioMed Amber and Dental LT Clear) with high precision, perfect molding, and fast printing speed compared to traditional materials. They found that BioMed Amber is more resistant to compression, while Dental LT clear is more resistant to stretching. Whether these new materials can be used as alternatives of titanium still needs evaluation. Furthermore, a study simulating the heat generation of an in vitro implant experiment showed that the involvement of a surgical guide produced more heat than the conventional implantation method; it suggests the necessity to further improve the cooling performance or heat generation features of the surgical guide in the future [21].

In a clinical perspective, patient-specific cutting guides are created to simplify the bone resection procedure, decrease possible errors and reduce the operation time [7,9,13,22]. Notably, Ma et al. [22] compared the accuracy and precision of image-based navigation versus individualized guides for distal radius osteotomy and showed statistically significant differences both in the standard deviation of ulnar variance error (2.0 mm for navigation vs. 0.6 mm for guides) and in the times required (705 s for navigation vs. 214 s for guides). Likewise, our 3D-printed titanium cutting guide shows a better performance in osteotomy accuracy in terms of the maximum 3D cutting deviation. Moreover, our 3D-printed titanium intraoral condylectomy cutting guide may serve as an optimal approach for certain cases, such as surgeons with less surgical navigation skills, limited surgical navigation instrument, and insufficient access and exposure of the deep condylar area. Since navigational operations must still overcome technology-related difficulties, such as systematic errors, registration errors, and operational errors due to the special anatomy, intraoperative hand–eye coordination and intraoperative instability of the mandible, the most favorable benefit of our cutting guide versus surgical navigation is that it does not require a registration process [11,12,13,22]. Thus, the surgeon can focus on performing the osteotomy and the saw is partly compelled by the guide during the osteotomy, which allows for less experienced surgeons to conduct this type of surgery in an accurate and fast manner. Nevertheless, our 3D-printed cutting guide does have some disadvantages compared with the intraoperative navigation technique. The positioning of the 3D-printed cutting guide can be inaccurate due to remaining soft tissue between the mandible and the cutting guide, as well as an insufficiently patient-specific shape of the guide. Because the 3D model of the bony structure was reconstructed based on a specific threshold CT value in the software, a discrepancy between the actual contour of the bone and the virtual one would exist if a too-high or too-low threshold CT value was chosen to reconstruct the mandible, which always results in the insufficiently patient-specific shape of the guide and a non-perfect fit during the surgery. If an intraoperative non-perfect fit was noticed or an unexpected shift occurred, the robustness of the printed titanium does not facilitate implementation of the virtual surgical plan and intraoperative changes of the osteotomy plane. Moreover, this technique is limited to patients who do not need condylar reconstruction or contouring; otherwise, an extraoral incision should be needed. Admittedly, the sample size of the present study was relatively limited by far and some important clinical data such as the surgical duration and blood volume were not recorded separately; more cases with long-term observation will be needed to achieve more clinical data in the future.

## 5. Conclusions

In summary, our results show that both 3D-printed cutting-guide-assisted and surgical-navigation-assisted intraoral condylectomy have a high accuracy and efficiency, while using a cutting guide can generate a relatively higher surgical accuracy. Moreover, the cutting guides do not need the extra registration process commonly used in surgical navigation and exhibit user-friendly features and simplicity for intraoral condylectomy, which represents a promising prospect in everyday clinical practice.

## Figures and Tables

**Table 1:** General clinical information of the patients.

Patient Number	Group	Age	Sex	Affected Side	Surgery *
1	Cutting guide group	23	Female	Right	Lefort I osteotomy + Right condylectomy+ Left SSRO
2	Cutting guide group	22	Female	Right	Lefort I osteotomy + Right condylectomy+ Left SSRO + Genioplasty
3	Cutting guide group	31	Female	Right	Lefort I osteotomy + Right condylectomy+ Left SSRO
4	Cutting guide group	24	Female	Right	Right condylectomy
5	Cutting guide group	26	Female	Right	Lefort I osteotomy + Right condylectomy+ Left SSRO + Genioplasty
6	Cutting guide group	34	Female	Right	Lefort I osteotomy + Right condylectomy+ Left SSRO
7	Cutting guide group	22	Female	Left	Lefort I osteotomy + Left condylectomy+ Right SSRO
8	Cutting guide group	23	Female	Left	Lefort I osteotomy + Left condylectomy+ Right SSRO + Genioplasty
9	Cutting guide group	30	Female	Right	Lefort I osteotomy + Right condylectomy+ Left SSRO
10	Cutting guide group	28	Female	Right	Lefort I osteotomy + Right condylectomy+ Left SSRO + Genioplasty
11	Navigation group	30	Female	Left	Left condylectomy
12	Navigation group	27	Male	Left	Lefort I osteotomy + Left condylectomy+ Right SSRO
13	Navigation group	18	Male	Right	Lefort I osteotomy+ Right condylectomy + Left SSRO + Genioplasty
14	Navigation group	23	Male	Right	Lefort I osteotomy+ Right condylectomy + Left SSRO
15	Navigation group	25	Female	Right	Lefort I osteotomy+ Right condylectomy + Left SSRO
16	Navigation group	24	Female	Right	Lefort I osteotomy + Right condylectomy+ Left SSRO
17	Navigation group	22	Female	Left	Lefort I osteotomy+ Left condylectomy + Right SSRO
18	Navigation group	22	Female	Left	Lefort I osteotomy + Left condylectomy+ Right SSRO + Genioplasty
19	Navigation group	20	Female	Right	Lefort I osteotomy + Right condylectomy+ Left SSRO
20	Navigation group	18	Female	Left	Lefort I osteotomy+ Left condylectomy + Right SSRO
21	Navigation group	21	Female	Right	Lefort I osteotomy+ Right condylectomy + Left SSRO

* Mandibular contour trimming was not listed in the surgical procedure.

**Table 2:** The asymmetry index of the landmarks Go, MF and Sg of the patients before and after the surgery.

	Chin Deviation			Chin Rotation			AI of Go			AI of MF			AI of Sg		
	Cutting Guide Group	Navigation Group	Significance	Cutting Guide Group	Navigation Group	Significance	Cutting Guide Group	Navigation Group	Significance	Cutting Guide Group	Navigation Group	Significance	Cutting Guide Group	Navigation Group	Significance
Pre-operation	8.61 ± 4.21	8.54 ± 4.77	NS	8.823 ± 2.67	8.98 ± 2.28	NS	17.64 ± 4.83	16.37 ± 3.77	NS	18.82 ± 4.69	19.31 ± 6.28	NS	11.83 ± 3.14	10.86 ± 4.43	NS
Post-operation	3.26 ± 2.62	3.03 ± 2.83	NS	4.28 ± 1.78	3.81 ± 1.82	NS	9.21 ± 2.76	7.13 ± 2.28	NS	8.83 ± 3.31	8.01 ± 3.48	NS	7.36 ± 2.79	6.69 ± 2.51	NS
Significance	*p* = 0.0058	*p* = 0.0034		*p* = 0.0004	*p* = 0.0001		*p* = 0.0012	*p* = 0.0005		*p* < 0.0001	*p* < 0.0001		*p* = 0.0391	*p* = 0.0438	

AI: Asymmetry index; NS: None significance.

## Data Availability

Not applicable.
